# Genome-Wide Mapping of DNA Methylation in Chicken

**DOI:** 10.1371/journal.pone.0019428

**Published:** 2011-05-05

**Authors:** Qinghe Li, Ning Li, Xiaoxiang Hu, Jinxiu Li, Zhuo Du, Li Chen, Guangliang Yin, Jinjie Duan, Haichao Zhang, Yaofeng Zhao, Jun Wang, Ning Li

**Affiliations:** 1 State Key Laboratory for Agrobiotechnology, China Agricultural University, Beijing, People's Republic of China; 2 Beijing Genomics Institute at Shenzhen, Shenzhen, People's Republic of China; 3 The Graduate University of Chinese Academy of Sciences, Beijing, People's Republic of China; Université Paris-Diderot, France

## Abstract

Cytosine DNA methylation is an important epigenetic modification termed as the fifth base that functions in diverse processes. Till now, the genome-wide DNA methylation maps of many organisms has been reported, such as human, Arabidopsis, rice and silkworm, but the methylation pattern of bird remains rarely studied. Here we show the genome-wide DNA methylation map of bird, using the chicken as a model organism and an immunocapturing approach followed by high-throughput sequencing. In both of the red jungle fowl and the avian broiler, DNA methylation was described separately for the liver and muscle tissue. Generally, chicken displays analogous methylation pattern with that of animals and plants. DNA methylation is enriched in the gene body regions and the repetitive sequences, and depleted in the transcription start site (TSS) and the transcription termination site (TTS). Most of the CpG islands in the chicken genome are kept in unmethylated state. Promoter methylation is negatively correlated with the gene expression level, indicating its suppressive role in regulating gene transcription. This work contributes to our understanding of epigenetics in birds.

## Introduction

DNA methylation is a stable epigenetic modification found in most of the eukaryotes that plays a crucial role in many biological processes, including gene expression regulation, gene imprinting and transposon silencing in mammals and plants [1], [2], [3]. In mammals, cytosine DNA methylation occurs mostly at the CpG dinucleotides except for the CpGs in CpG islands [4]. DNA methylation is unevenly distributed in the genome, the heterochromatin region, transposons and repetitive sequences are usually hypermethylated, and the 5′ and 3′ flanking regions of genes are methylated at a relatively low level compared with the gene body regions [3], [5], [6]. Although many genome-wide DNA methylation profiles and their functional analysis have been reported, there is little knowledge about the DNA methylation patterns in birds [5], [6], [7], [8], [9].

There are many approaches to decipher a genome-wide DNA methylation profile, including methylated DNA immunoprecipitation-sequencing/chip (meDIP-seq/chip), bisulfite-sequencing (bis-seq) and some enzyme digestion based techniques. MeDIP uses an antibody which can specifically recognizes methylated cytosines and pulls down the methylated fractions, MeDIP-chip was used to provide the first comprehensive DNA methylation map of an entire Arabidopsis thaliana genome [3].

The gold standard to determine the DNA methylome is genome-wide bisulfite sequencing, which firstly converts all the unmethylated cytosines into uracil while left the methylated cytosines unchanged by sodium bisulfite under denaturing conditions, which can be distinguished subsequently by sequencing [10]. Despite its high resolution, genome-wide bis-seq remains a high cost and time-consuming method for DNA methylome study. Many studies showed that meDIP combined with high-throughput sequencing or chip could be considered as a method that can reflect the relative methylation state of a genome [3], [11], [12].

The chicken (*Gallus gallus*) is an important animal model that bridges the mammals and vertebrates in evolution and has long been used as a model species for the study of embryology, immunology, behavior and reproduction [13]. The red jungle fowl is believed to be the ancestor of the domestic chicken, and chicken are thought to have been domesticated about 8,000 years ago, in South-East Asia [14], [15]. Among numerous chicken breeds raised by many years of adaptation and breeding, the avian broiler shows good performance on growth, muscle yield, feed efficiency and disease resistance, making it a common chicken breed for meat production [16].

To study the global DNA methylation patterns in the chicken genome, we generated the DNA methylomes of the red jungle fowl and avian broiler by meDIP-seq using Illumina Genome Analyzer II. Liver and muscle tissue were selected for methylation analysis due to their biological and economical importance. Our results provided the first insight into DNA methylation landscape in birds.

## Results

### Global mapping of DNA methylation in chicken

To decipher the genome-wide DNA methylome of the chicken, we dissected liver and muscle tissues from 7-day-old chickens, we immunoprecipitated sheared genomic DNA with an antibody which specifically recognizes 5-methylcytosine and sequenced the enriched methylated DNA of the liver and muscle tissue of the red jungle fowl and the avian broiler (RJF liver, RJF muscle, AA liver and AA muscle with Illumina Genome Analyzer II. MeDIP-Seq reads were aligned using Maq [17] and only the uniquely mapped reads were used in scanning the methylation peak (regions with sequencing tags more than 10 and *p* value<10^−5^) with MACS [18]. A range of 17,202,074 to 27,501,760 raw reads were generated for the four samples respectively and more than 85% of the reads were mapped and about 65% of the reads in each sample were uniquely mapped to the chicken genome in each sample (Table 1).

**Table 1:** MeDIP-Seq Illumina GA data.

	Total meDIP-Seq data	Percentage of mapped reads in total reads	Percentage of unique mapped reads	Percentage of unmapped reads
AA liver	20,445,577	85.47%	63.94%	14.53%
AA muscle	18,203,752	88.52%	67.43%	11.48%
RJF liver	27,501,760	89.38%	67.66%	10.62%
RJF muscle	17,202,074	87.73%	65.51%	12.27%

**Figure 1:**
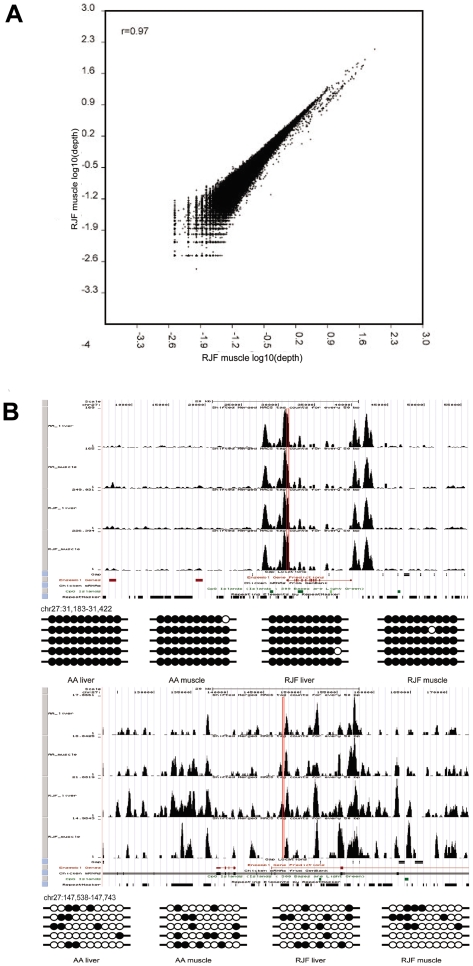
Global mapping of DNA methylation in chicken. **A**. Scatter plot showing the correlation between two independent meDIP-Seq experiments with RJF muscle used for DNA methylation assay. **B**. Snapshot of meDIP-Seq data from genome-wide DNA methylation analysis. The DNA methylation signal is shown with the sequencing tags number along the chromosome. Gene region is shown with the red boxes. Methylation patterns of selected high methylated region and low methylated region were detected by bisulfite sequencing.

The technical reproducibility was assessed by two independent meDIP experiments and sequencing performances for RJF muscle. Each sample was sequenced by Solexa for one lane: the correlation coefficient (Pearson's r) of these two samples was 0.97 (*p*<0.0001), which indicates our approach is highly reproducible (Fig. 1A). We carried out bis-seq for 8 randomly selected regions in the chicken genome, and the bis-seq results were feckly consistent with the meDIP-seq results (Fig. 1B, Fig S1 and Fig S2). These results indicated that our methylation data obtained by meDIP-seq was reliable.

### Distribution of DNA methylation around the transcription start sites (TSS)

**Figure 2:**
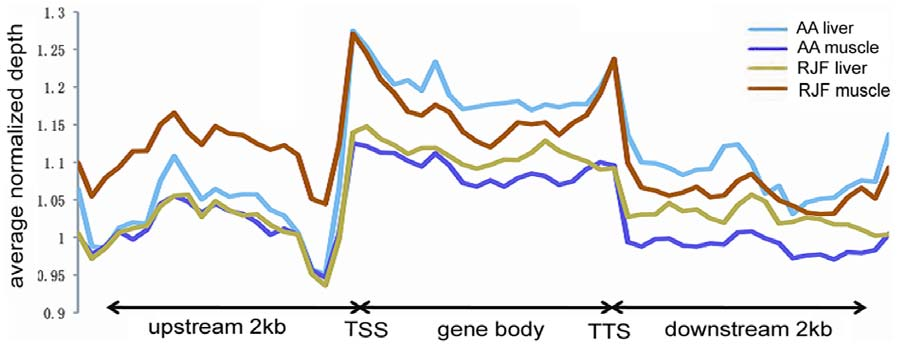
DNA methylation distribution in gene flanking and coding regions. DNA methylation profile in gene region was calculated by the tags that were aligned on unique locus in genome. The gene region was defined as the whole regions that contained 2 kb region upstream of the TSS, gene body from TSS to TTS and 2 kb region downstream of the TTS. In upstream and downstream 2 kb regions, the regions were split into 20 non-overlap windows and the average alignment depth was calculated for each window. In gene body, each gene was split into 20 equal windows and the average alignment depth was calculated for each window. Y-axis is the average of normalized depth for each window.

First we analyzed the distribution of DNA methylation in the 2 kb region upstream of the transcription start sites, the gene body and the 2 kb region downstream of the transcription termination site. Generally, gene body regions show a higher level of DNA methylation than the 5′ and 3′ flanking regions of genes [3]. The genome region around TSS is crucial for gene expression regulation. In chicken, DNA methylation level decreased dramatically before the TSS and increased sharply towards the gene body regions and stayed at a plateau until the 3′ end of the gene body (Fig. 2). Previous studies have demonstrated that DNA methylation in the gene body regions impeded transcription elongation in *Neusprora crassa*, *Arabidopsis thaliana* and mammalian cells [5], [19], [20]. The hypermethylation of the gene body regions in the chicken genome further indicates that this is probably a mechanism for regulation of gene expression that is conserved among species.

### DNA methylation in CpG islands and repetitive sequences

CpG islands were reported to be relatively lowly methylated [8]. We first evaluated the total number of the CpG islands in the chicken genome with the criteria of length >200 bp, G+C content >50% and CpG observed to expected >0.6 [21]. A total number of 20224 CpG islands were identified in the chicken genome. Subsequently, we estimated the methylation status of the CpG islands: CpG islands that overlapped with the methylation peaks were termed as methylated CpG islands. Of all the CpG islands in the chicken genome, about 9.1% were methylated in AA liver, 7.3% in AA muscle, 5.7% in RJF liver and 13.1% in RJF muscle of the CpG islands were methylated in the chicken genome (Table 2). Overall, most CpG islands were unmethylated in chicken, CpG islands in the RJF-liver were least likely to be methylated, with the greatest proportion of methylated CpG islands in RJF muscle. The 5′ end of the gene is important for the gene expression regulation and the methylation of the 5′ end is usually suppressive for gene expression. Our results showed that about 10 percents of the methylated CpG islands were in this region in the chicken genome.

**Table 2:** Summary of methylated CpG islands in the liver and muscle tissue of the red jungle fowl and avian broiler.

Sample	5′ end of a gene	3′ end of a gene	Exon	Intron	Intergenic	Total methylated CGIs	Total CGIs	Methylated (%)
AA liver	168	63	239	251	1716	1850	20224	9.1
AA muscle	171	45	207	215	1371	1472	20224	7.3
RJF liver	128	30	150	158	1083	1156	20224	5.7
RJF muscle	362	69	430	461	2289	2657	20224	13.1

The density of interspersed repeats is less than 9% in chicken genome, which is much lower than that in mammalian genomes [22]. In accordance with this phenomenon, less than 10% of the uniquely mapped meDIP-seq reads in chicken belonged to the repeat sequences annotated by UCSC (Fig. S3). The predominant type of interspersed repeat in the chicken genome, chicken repeat 1 (CR1), accounted for about 60% of the total methylated repeat sequences (Table S1).

### Promoter DNA methylation and gene expression level

**Figure 3:**
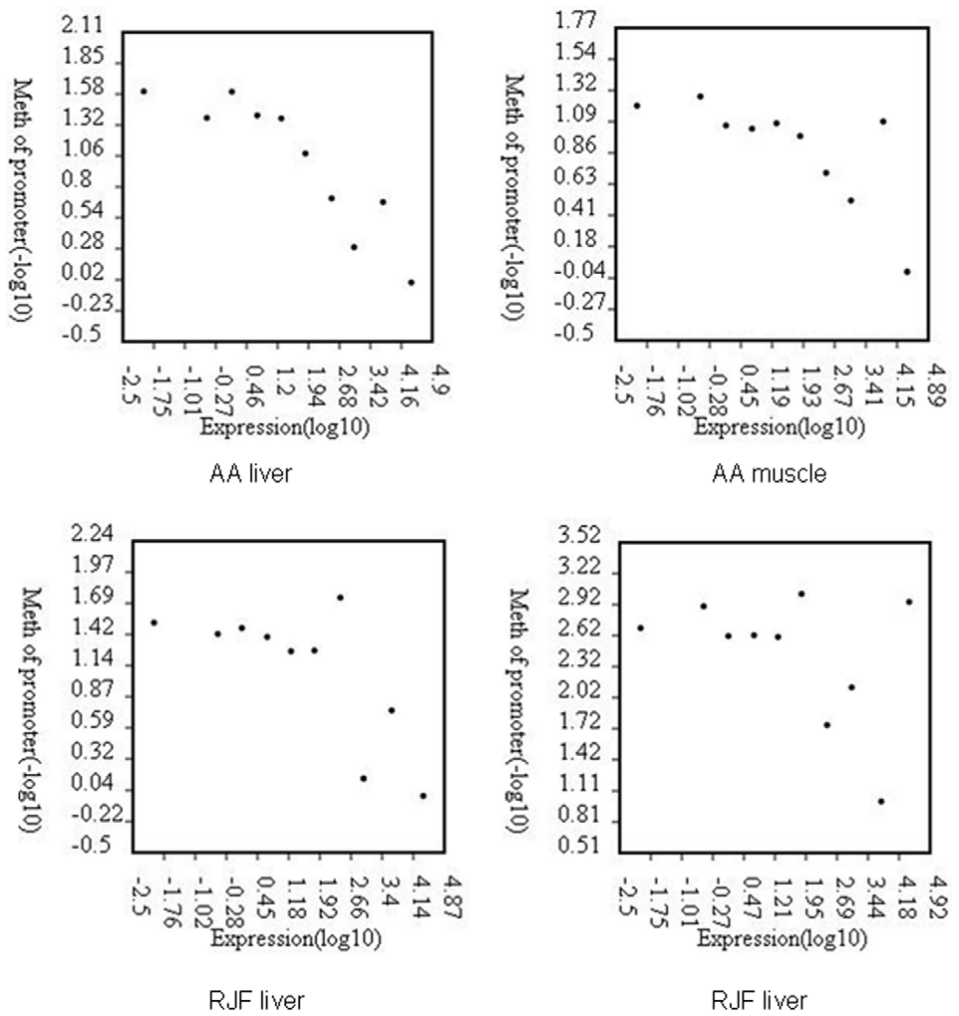
Relationship between promoter DNA methylation and gene expression level in chicken. Genes were divided into 10 intervals according to expression levels. DNA methylation level was measured by the log ratio of the p value of the methylation peaks, with each point representing the mean expression level and the relative methylation level.

Most of the promoter regions are associated with CpG islands and are lowly methylated. Promoter DNA methylation always causing a compact chromatin structure and is recognized as repressive signal for gene transcription. By RNA-seq we got the gene expression profiles for each of RJF liver, RJF muscle, AA liver and AA muscle. In the present study we divided genes into ten groups based on expression levels, from the lowest 10% and to the highest 10%. Here we defined the genomic regions 2 kb upstream and downstream of the TSS as the proximal promoter, and used the p value of the methylation peaks for the measurement of methylation level. We observed that gene expression level is negatively correlated with DNA methylation in the proximal promoter regions in the AA liver (Fig. 3), AA muscle, RJF liver (AA liver, r = −0.90, p<0.01; AA muscle, r = −0.73, p<0.05; RJF liver, r = −0.72, p<0.05), while there was a little undulation in RJF muscle (r = −0.43, p = 0.2).

### Distribution of highly methylated regions and methylated genes enrichment in chicken genome

MeDIP-seq is more suitable for analysis for DNA methylation status of the regions that are heavily methylated in the genome. There were, in total, 25974 HMRs in AA-liver, 23965 HMRs in AA-muscle, 20654 HMRs in RJF-liver and 47349 HMRs in RJF-muscle. Using the 5′ and 3′ sequence information annotated for the chicken genome in UCSC and defining intergenic regions as sequences between the annotated 3′ and 5′ ends of the genes, we observed that 1.2% to 1.5% of the HMRs were at the 5′ end of genes, 0.7% to 1.2% of the HMRs were at the 3′ end, 3.7% to 5.7% of the HMRs were in the exons and 9.5% to 11.4% of the HMRs were in the introns in the four samples, most of the HMRs fell into the intergenic regions (Table 3).

**Table 3:** Distribution of HMRs in the chicken genome.

	Total peak number	5′ end of gene	3′ end of gene	exon	intron	intergenic
AA liver	25974	323	297	1489	2775	22534
AA muscle	23965	291	178	896	2280	21194
RJF liver	20654	325	248	1022	2191	18288
RJF muscle	47349	712	553	2387	5390	41482

As a tool for gene expression regulation, DNA methylation is an important epigenetic marker that shows stability and flexibility between different generations. To investigate the function distribution of the methylated genes, we performed the gene ontology analysis (*p*<0.01) for the methylated genes in each of AA liver, AA muscle, RJF liver and RJF muscle. Methylated genes were defined as genes overlapped with HMRs in promoter regions and gene body. In AA liver, the methylated genes were enriched in the functions of zinc ion and metal ion binding, protein phosphorylation, *etc* (Table S2). In AA muscle the methylated genes were enriched in gamma-aminobutyric acid secretion, neuronal action potential propagation, synaptic transmission, *etc* (Table S2). In RJF liver, methylated genes enriched in actomyosin structure organization, phospholipase inhibitor activity, and tropomyosin binding, *etc* (Table S2), while in RJF muscle methylated genes showed enrichment in protein phosphorylation, calcium ion binding and so on (Table S2).

## Discussion

Here we report the use of meDIP-seq to determine the genome-wide DNA methylation patterns in liver and muscle tissues of the red jungle fowl and the avian broiler. Our results indicated that the chicken shows analogous DNA methylation patterns to those of mammals and plants [3], [8], [23]. In the chicken, the repetitive sequences are hypermethylated while most of the CpG islands remain hypomethylated, gene body regions show a much higher level of DNA methylation than the 5′ and 3′ flanking regions.

Genomic imprinting is a germline specific epigenetic modification which caused allelic specific expression pattern of parental genes [24]. Imprinted genes are found in eutherian mammals, marsupials and flowering plants but there is no report of this phenomenon in birds [25]. The genes *Mpr/Igf2r*, *Igf2*, *Ascl2/Mash2* and *Ins2* have been shown to be imprinted in mammals but were all found to be expressed biallelically in birds [24], [26], [27], [28]. In the present study we tried to find any indication of imprinting in chicken, we analyzed the putative differentially methylated regions (DMRs) because the majority of imprinted genes identified so far have differentially methylated alleles [25], [29], and as expected, we did not identify any experimentally demonstrable DMRs in the chicken genome (data not shown). Our results further indicated the absence of genomic imprinting in birds and the uniqueness of gene imprinting in viviparous animals and plants.

In conclusion, we have generated the first, to our knowledge, DNA methylome for a bird species. We found meDIP-seq was able to provide the DNA methylation landscape in chicken, and the methylated genomic regions with meDIP–seq enrichment could be validated by bis-seq. These DNA methylome maps will be useful for further studies on epigenetic gene regulation in chicken and other birds. Xu *et al* reported the overall methylation differences between different tissues and strains of chicken [30], which provided the first attempt to elucidate the DNA methylation variations between chicken breeds with heterogeneous genetic background. But due to the lack of enough biological replicates, it was hard for us to carry out comprehensive analysis on methylation variations between different chicken breeds. The epigenetic system existing in the chicken genome lays a foundation for studying the involvement of epigenetic modifications in chicken domestication,and more systemic analysis of DNA methylation of different chicken breeds are needed to elucidate this problem.

## Materials and Methods

### Animals

Two 7-day-old female chickens were utilized in this experiment, one red jungle fowl and one avian broiler. The chickens were fed with the same diet and sacrificed according to local standards of animal welfare issues. The study was approved by animal welfare committee of China Agricultural University with approval number XK257. Liver and muscle tissues taken from each animal were flash frozen in liquid nitrogen and then stored at −80°C.

### DNA preparation and meDIP-seq

DNA was isolated by phenol-chloroform extraction. DNA was sonicated at 40 w for 8 times with pause for 1 min on ice (Sonics, VC130PB), and DNA fragments ranging from 200–350 bp were retracted by gel excision with gel extraction kit (Qiagen,). The recovered DNA was first 5′ and 3′ end blunting, phosphorylating and repairing by T4 Polynucleotide Kinase and T4 DNA Polymerase (NEB). After addition of an ATP in the 3′ end, an Illumina sequencing primer adapter was ligated to the DNA using the Quick Ligation™ Kit (Qiagen). DNA was recovered by MinElute® PCR Purification Kit (Qiagen) and used for meDIP. Our meDIP method was modified from previous study [3]. For each sample, we incubated 4 µg denatured DNA with 32 µg anti-5-methylcytosine mouse monoclonal antibody (Calbiochem) in 400 µl IP buffer (10 mM Tris-HCl, pH 7.5, 280 mM NaCl, 1 mM EDTA) at 4°C for 5.5 hr. Then we added 100 µl Dynabeads Protein G and Protein A (Dynal) to the mix and incubated at 4°C for 5.5 hr. The following step was the same as in the method described by Xiaoyu Zhang [3]. After meDIP, the DNA was divided into three fractions: the unbound, washed and bound fractions. DNA in the bound fraction from qualifying meDIP experiment was PCR amplified with sequencing primers provided by Illimina using Phusion™ High-Fidelity PCR Master Mix (Finnzymes) under the following conditions: 3 min at 98°C; followed by 18 cycles of 98°C for 15 s, 65°C for 30 s, 72°C for 20 s; and a final extension for 5 min. PCR products were recovered and used for Solexa sequencing.

### Public data used and Gene Ontology annotation

The chicken reference genome (galGal3), together with annotation of repeats, was downloaded from the UCSC database (http://hgdownload.cse.ucsc.edu/goldenPath/galGal3). Gene information was downloaded from the public FTP site of Ensembl (ftp://ftp.ensembl.org/pub) in October 2008. The information about GO terms was downloaded from the UniProtKB-GOA database. We select random samples of *Nf* different genes at each iteration and compute Fisher's exact test p-values for over-representation of the selected genes in all GO biological categories. GO terms with p<0.05 were considerd as significant enriched.

### MeDIP-Seq sequence alignments and data analysis

35 bp sequencing reads and resulting FASTQ files were aligned to the chicken reference genome (galGal3) by the open-source aligner the Mapping and Assembly with Qualities (MAQ). 2 bp mismatches were allowed and retained uniquely mapped reads for further analysis. The output of the MAQ was also converted to browser extensible data (BED) files for viewing the data in the UCSC genome browser. Model-based Analysis of ChIP-Seq (MACS) to was used to scan the methylated peaks in the genome. Subsequently, the genes with DNA methylation peaks were employed for GO analysis.

### Bisulfite-sequencing

DNA was sonicated to 500–1000 bp long and recovered by MinElute® PCR Purification Kit (Qiagen). 2 µg sonicated DNA bisulfite treated with EZ DNA methylation-goldTM kit (Zymo Research). To examine the methylation status of specific regions, we carried out semi-nested PCR under the following conditions. The first round of amplification comprised: 5 min at 94°C; 20 cycles of 94°C for 30 s, 50°C for 30 s and 72°C for 30 s, with a final extension at 72°C for 5 min. The second round of amplification comprised: 5 min at 94°C; 35cycles of 94°C for 30 s, appreciate Tm for 30 s and 72°C for 30 s, with a final extension at 72°C for 5 min. The PCR products were gel-purified using a Gel Extraction Kit (Qiagen), cloned into the pMD™ 19-T Vector (Takara) and sequenced.

### RNA-Seq

A piece of tissue was ground in liquid nitrogen, total RNA was extracted with TRIZOL® Reagent (Invitrogen). 30 µg total RNA was digested with RNase-Free DNase I (NEB) for 15 min at 37°C, phenol-chloroform extraction and ethanol precipitation to remove DNA contamination. The concentration and quality of RNA were assessed by Agilent 2100. 2 ug total RNA was used in library construction. The mRNAs were isolated and reverse transcribed into double-stranded cDNA on magnetic beads covered with oligo d(T), the cDNA was digested with Nla *III* and ligated to Illumina adapter containing a recognition site of Mme *I*. Following Mme *I* digestion, a second Illumina adapter was ligated. Tags closed to the 3′ terminus of the mRNA were enriched by a 15 cycles PCR. The PCR products at 85 bp DNA band was excised and purified for cluster generation and sequencing analysis. The sequences obtained were mapped onto the refSeq database. Sequences uniquely mapped to refseq genes were used for subsequent analysis. All libraries were normalized to 1 M sequences according to clean sequences.

The sequencing data from this study have been submitted to the NCBI Gene Expression Omnibus (http://www.ncbi.nlm.nih.gov/geo) under accession no. GSE21167, GSE21169 and GSE21170.

## Supporting Information

Figure S1 **Bis-seq results of 4 methylation peak regions.**
(TIF)

Figure S2 **Bis-seq results of 2 regions without methylation peak.**
(TIF)

Figure S3 **The component percentage of mapped meDIP-seq reads.** All of thuniquely mapped reads were classified into four types: the reads that were uniquely mapped into CpG islands (blue), genes bodies from transcript starting site to transcript ending site (red), repeats which were annotated by Repeat Masker and published on UCSC (green) , genome except for CpG Islands, gene body and repeats.
(TIF)

Table S1 **The component percentage of the uniquely mapped reads in different repeat types.**
(DOC)

Table S2 **Gene ontology analysis results for proximal promoter methylated genes**
(DOC)

## References

[1] Goll MG, Bestor TH (2005). Eukaryotic cytosine methyltransferases.. Annu Rev Biochem.

[2] Bird A (2002). DNA methylation patterns and epigenetic memory.. Genes Dev.

[3] Zhang X, Yazaki J, Sundaresan A, Cokus S, Chan SW (2006). Genome-wide high-resolution mapping and functional analysis of DNA methylation in arabidopsis.. Cell.

[4] Weber M, Davies JJ, Wittig D, Oakeley EJ, Haase M (2005). Chromosome-wide and promoter-specific analyses identify sites of differential DNA methylation in normal and transformed human cells.. Nat Genet.

[5] Zilberman D, Gehring M, Tran RK, Ballinger T, Henikoff S (2007). Genome-wide analysis of Arabidopsis thaliana DNA methylation uncovers an interdependence between methylation and transcription.. Nat Genet.

[6] Gehring M, Bubb KL, Henikoff S (2009). Extensive demethylation of repetitive elements during seed development underlies gene imprinting.. Science.

[7] Hsieh TF, Ibarra CA, Silva P, Zemach A, Eshed-Williams L (2009). Genome-wide demethylation of Arabidopsis endosperm.. Science.

[8] Eckhardt F, Lewin J, Cortese R, Rakyan VK, Attwood J (2006). DNA methylation profiling of human chromosomes 6, 20 and 22.. Nat Genet.

[9] Lister R, O'Malley RC, Tonti-Filippini J, Gregory BD, Berry CC (2008). Highly integrated single-base resolution maps of the epigenome in Arabidopsis.. Cell.

[10] Frommer M, McDonald LE, Millar DS, Collis CM, Watt F (1992). A genomic sequencing protocol that yields a positive display of 5-methylcytosine residues in individual DNA strands.. Proc Natl Acad Sci U S A.

[11] Li N, Ye M, Li Y, Yan Z, Butcher LM (2010). Whole genome DNA methylation analysis based on high throughput sequencing technology.. Methods.

[12] Ruike Y, Imanaka Y, Sato F, Shimizu K, Tsujimoto G (2010). Genome-wide analysis of aberrant methylation in human breast cancer cells using methyl-DNA immunoprecipitation combined with high-throughput sequencing.. BMC Genomics.

[13] Burt D, Pourquie O (2003). Genetics. Chicken genome–science nuggets to come soon.. Science.

[14] Fumihito A, Miyake T, Sumi S, Takada M, Ohno S (1994). One subspecies of the red junglefowl (Gallus gallus gallus) suffices as the matriarchic ancestor of all domestic breeds.. Proc Natl Acad Sci U S A.

[15] Rubin CJ, Lindberg J, Fitzsimmons C, Savolainen P, Jensen P (2007). Differential gene expression in femoral bone from red junglefowl and domestic chicken, differing for bone phenotypic traits.. BMC Genomics.

[16] Rubin CJ, Zody MC, Eriksson J, Meadows JR, Sherwood E (2010). Whole-genome resequencing reveals loci under selection during chicken domestication.. Nature.

[17] Li H, Ruan J, Durbin R (2008). Mapping short DNA sequencing reads and calling variants using mapping quality scores.. Genome Res.

[18] Zhang Y, Liu T, Meyer CA, Eeckhoute J, Johnson DS (2008). Model-based analysis of ChIP-Seq (MACS).. Genome Biol.

[19] Lorincz MC, Dickerson DR, Schmitt M, Groudine M (2004). Intragenic DNA methylation alters chromatin structure and elongation efficiency in mammalian cells.. Nat Struct Mol Biol.

[20] Rountree MR, Selker EU (1997). DNA methylation inhibits elongation but not initiation of transcription in Neurospora crassa.. Genes Dev.

[21] Gardiner-Garden M, Frommer M (1987). CpG islands in vertebrate genomes.. J Mol Biol.

[22] consortium Icgs (2004). Sequence and comparative analysis of the chicken genome provide unique perspectives on vertebrate evolution.. Nature.

[23] Cokus SJ, Feng S, Zhang X, Chen Z, Merriman B (2008). Shotgun bisulphite sequencing of the Arabidopsis genome reveals DNA methylation patterning.. Nature.

[24] Yokomine T, Shirohzu H, Purbowasito W, Toyoda A, Iwama H (2005). Structural and functional analysis of a 0.5-Mb chicken region orthologous to the imprinted mammalian Ascl2/Mash2-Igf2-H19 region.. Genome Res.

[25] Reik W, Walter J (2001). Genomic imprinting: parental influence on the genome.. Nat Rev Genet.

[26] Killian JK, Byrd JC, Jirtle JV, Munday BL, Stoskopf MK (2000). M6P/IGF2R imprinting evolution in mammals.. Mol Cell.

[27] Nolan CM, Killian JK, Petitte JN, Jirtle RL (2001). Imprint status of M6P/IGF2R and IGF2 in chickens.. Dev Genes Evol.

[28] O'Neill MJ, Ingram RS, Vrana PB, Tilghman SM (2000). Allelic expression of IGF2 in marsupials and birds.. Dev Genes Evol.

[29] Anway MD, Cupp AS, Uzumcu M, Skinner MK (2005). Epigenetic transgenerational actions of endocrine disruptors and male fertility.. Science.

[30] Xu Q, Zhang Y, Sun D, Wang Y, Yu Y (2007). Analysis on DNA methylation of various tissues in chicken.. Anim Biotechnol.

