# Supplementary figures and images for: Genome-Wide Mapping of DNA Methylation in Chicken
Source: PLoS One. 2011 May 5;6(5):e19428. doi: 10.1371/journal.pone.0019428 (PMC3088676; doi:10.1371/journal.pone.0019428)

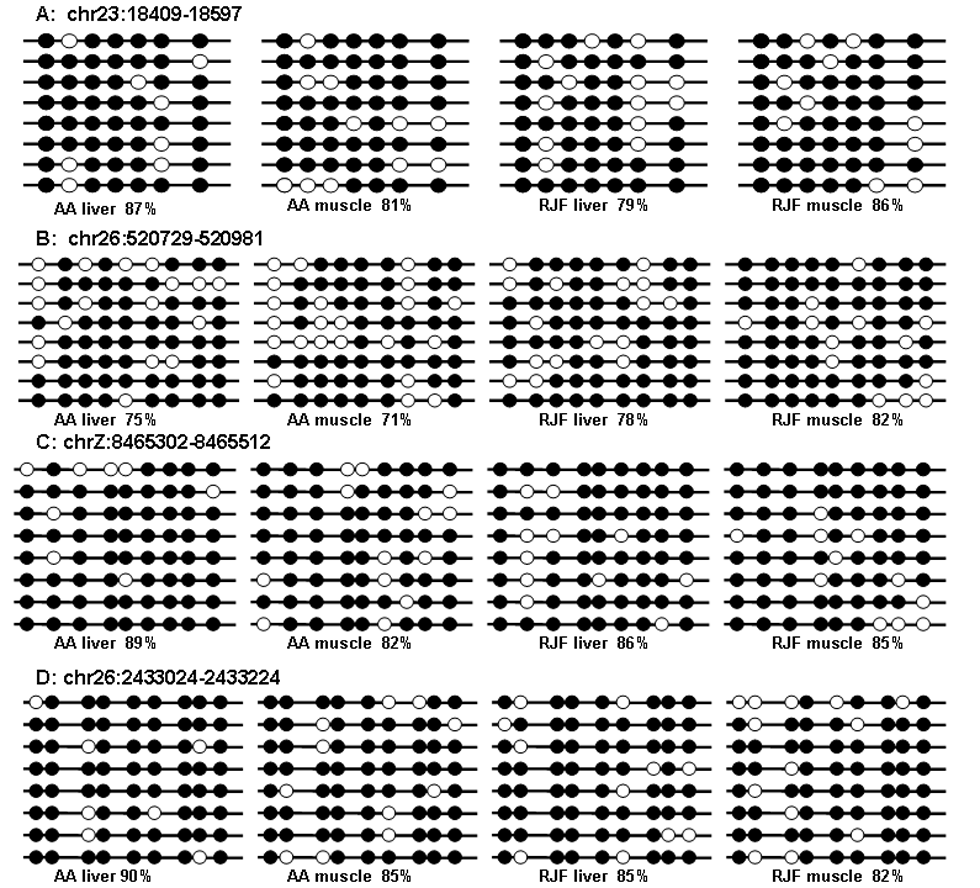

Supplement: Figure S1 — Bis-seq results of 4 methylation peak regions. (TIF) [file pone.0019428.s001.tif]

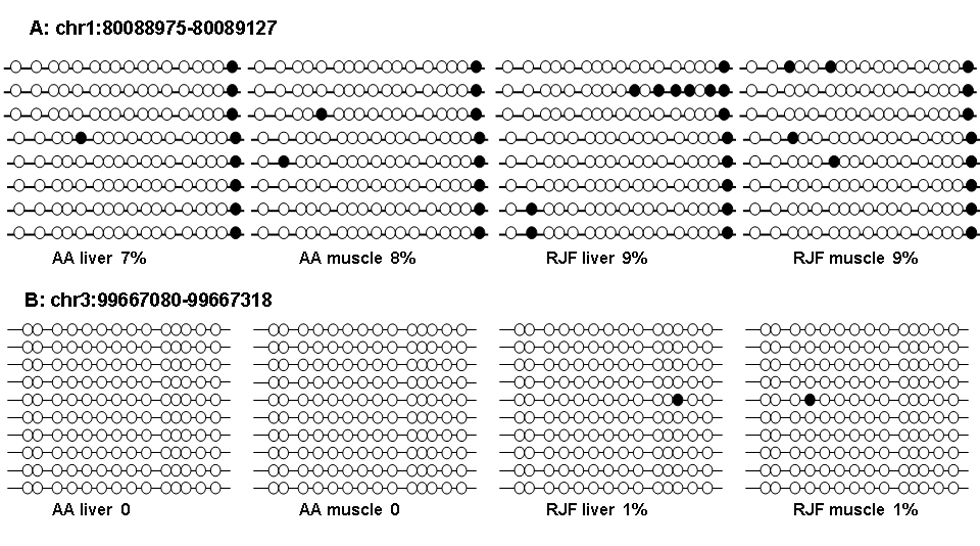

Supplement: Figure S2 — Bis-seq results of 2 regions without methylation peak. (TIF) [file pone.0019428.s002.tif]

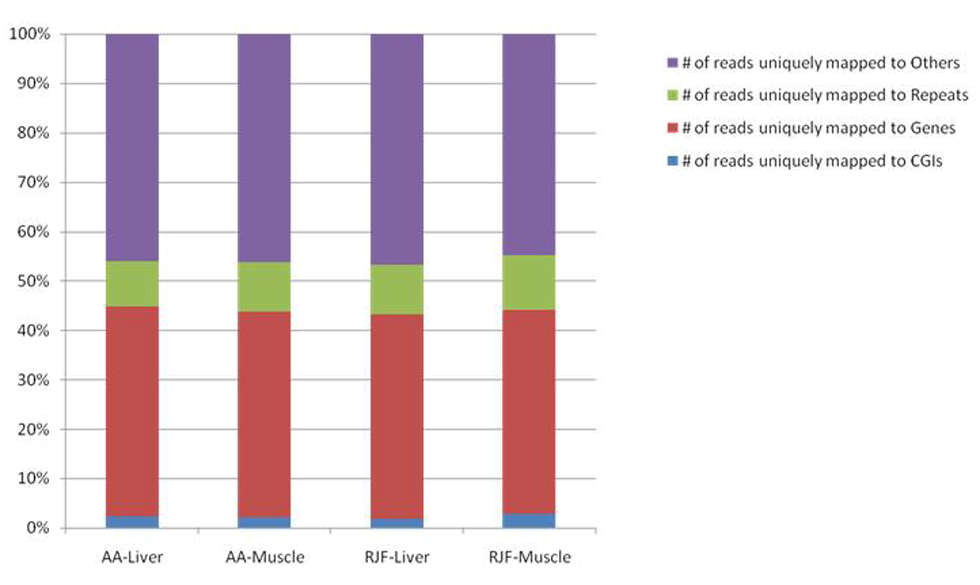

Supplement: Figure S3 — The component percentage of mapped meDIP-seq reads. All of thuniquely mapped reads were classified into four types: the reads that were uniquely mapped into CpG islands (blue), genes bodies from transcript starting site to transcript ending site (red), repeats which were annotated by Repeat Masker and published on UCSC (green) , genome except for CpG Islands, gene body and repeats. (TIF) [file pone.0019428.s003.tif]
