# Supplementary material for: Genome-Wide Mapping of DNA Methylation in Chicken
Source: PLoS One. 2011 May 5;6(5):e19428. doi: 10.1371/journal.pone.0019428 (PMC3088676; doi:10.1371/journal.pone.0019428)
Supplement: Table S1 — The component percentage of the uniquely mapped reads in different repeat types. (DOC) [file pone.0019428.s004.doc]

Table S1 The component percentage of the uniquely mapped reads in different repeat types.

| sample  repeat type | AA liver (%) | AA muscle (%) | RJF liver (%) | RJF muscle (%) |
| --- | --- | --- | --- | --- |
| DNA/charlie | 1.15 | 1.01 | 0.99 | 1.01 |
| DNA/mariner | 5.08 | 7.03 | 7.37 | 5.50 |
| LINE/CR1 | 58.76 | 64.26 | 61.46 | 64.64 |
| LINE/L2 | 0 | 0 | 0 | 0 |
| Low complexity | 0.99 | 0.69 | 0.7 | 0.76 |
| LTR/ERV1 | 1.12 | 0.92 | 1.03 | 0.95 |
| LTR/ERVK | 0.97 | 0.56 | 0.56 | 0.68 |
| LTR/ERVL | 10 | 10.53 | 10.19 | 10.08 |
| Satellite | 3.22 | 1.97 | 2.33 | 2.45 |
| Satellite/macro | 0.67 | 0.52 | 0.49 | 0.67 |
| Satellite/w chromosome | 13.38 | 7.43 | 11.15 | 8.33 |
| Simple repeat | 4.66 | 5.08 | 3.74 | 4.94 |
| SINE/MIR | 0 | 0 | 0 | 0 |
| Unknown | 0 | 0 | 0 | 0 |
