# Supplementary material for: Genome-Wide Mapping of DNA Methylation in Chicken
Source: PLoS One. 2011 May 5;6(5):e19428. doi: 10.1371/journal.pone.0019428 (PMC3088676; doi:10.1371/journal.pone.0019428)
Supplement: Table S2 — Gene ontology analysis results for proximal promoter methylated genes (DOC) [file pone.0019428.s005.doc]

Table S2. Gene ontology analysis results for proximal promoter methylated genes

| **Sample** | **Gene Ontology (GO)** | **P-value for**  **enrichment** |
| --- | --- | --- |
| **AA liver** | zinc ion binding | 8.40E-11 |
|  | metal ion binding | 1.52E-08 |
|  | protein amino acid phosphorylation | 0.00028 |
|  | galactosyltransferase activity | 0.00044 |
|  | protein serine/threonine/tyrosine kinase activity | 0.0013 |
|  | vocal learning | 0.0013 |
|  | righting reflex | 0.0013 |
|  | skeletal muscle tissue development | 0.0017 |
|  | calmodulin-dependent protein kinase activity | 0.0026 |
|  | calmodulin binding | 0.0039 |
|  | cerebellum development | 0.0043 |
|  | post-embryonic development | 0.0045 |
|  | protein amino acid ADP-ribosylation | 0.0064 |
|  | nucleic acid binding | 0.0076 |
|  | smooth muscle tissue development | 0.0088 |
| **AA muscle** | gamma-aminobutyric acid secretion | 1.16E-05 |
|  | neuronal action potential propagation | 1.16E-05 |
|  | synaptic transmission, glutamatergic | 1.16E-05 |
|  | cAMP metabolic process | 1.16E-05 |
|  | muscle fiber development | 1.16E-05 |
|  | Peyer's patch development | 2.31E-05 |
|  | adult walking behavior | 0.00010 |
|  | regulation of membrane potential | 0.00014 |
|  | neuromuscular junction development | 0.00017 |
|  | T cell receptor signaling pathway | 0.00017 |
|  | gamma-aminobutyric acid signaling pathway | 0.00021 |
|  | spleen development | 0.00035 |
| **AA muscle (continued)** | voltage-gated calcium channel complex | 0.00040 |
|  | thymus development | 0.00040 |
|  | cellular calcium ion homeostasis | 0.00046 |
|  | voltage-gated calcium channel activity | 0.00052 |
|  | calcium ion transport | 0.0019 |
|  | GTPase activator activity | 0.0040 |
| **RJF liver** | actomyosin structure organization | 6.09E-06 |
|  | phospholipase inhibitor activity | 0.0030 |
|  | tropomyosin binding | 0.0030 |
|  | positive regulation of vesicle fusion | 0.0030 |
|  | fibrinolysis | 0.0030 |
|  | actin binding | 0.0046 |
| **RJF muscle** | protein amino acid phosphorylation | 6.06E-15 |
|  | calcium ion binding | 7.64E-12 |
|  | transferase activity, transferring phosphorus-containing groups | 7.99E-12 |
|  | protein serine/threonine kinase activity | 3.53E-11 |
|  | ATP binding | 6.90E-08 |
|  | homophilic cell adhesion | 3.31E-07 |
|  | protein tyrosine/serine/threonine phosphatase activity | 2.42E-05 |
|  | nucleotide binding | 4.59E-05 |
|  | protein amino acid dephosphorylation | 0.00021 |
|  | intracellular signal transduction | 0.00063 |
|  | peptidyl-serine phosphorylation | 0.00069 |
| **RJF muscle (continued)** | regulation of mitotic metaphase/anaphase transition | 0.00069 |
|  | positive regulation of axon extension involved in regeneration | 0.00069 |
|  | calmodulin-dependent protein kinase activity | 0.0014 |
|  | positive regulation of proteasomal ubiquitin-dependent protein catabolic process | 0.0023 |
|  | voltage-gated potassium channel complex | 0.0025 |
|  | anaphase-promoting complex | 0.0034 |
|  | mechanoreceptor differentiation | 0.0034 |
|  | voltage-gated potassium channel activity | 0.0054 |
|  | phosphoinositide phospholipase C activity | 0.0062 |
|  | neurotrophin receptor activity | 0.0062 |
|  | calcium-dependent phospholipid binding | 0.00623 |
|  | acid-amino acid ligase activity | 0.0074 |
|  | ubiquitin-protein ligase activity | 0.0098 |
